# Protocol for the HALDI study—conceptual framework for investigating health and living conditions in an arctic area of Sweden with a multiethnic population

**DOI:** 10.1080/07853890.2025.2537914

**Published:** 2025-07-28

**Authors:** Katarina Nägga, Per Axelsson, Christina Storm Mienna

**Affiliations:** ^a^ Department of Geriatrics and Palliative Medicine, and Department of Health, Medicine and Caring Sciences, Linköping University, Linköping, Sweden; ^b^ Várdduo – Centre for Sámi Research, Umeå University, Umeå, Sweden; ^c^ Department of Historical, Philosophical and Religious studies, Umeå University, Umeå, Sweden; ^d^ Centre for Demographic and Ageing Research, Umeå University, Umeå, Sweden; ^e^ Department of Odontology, Orofacial pain and Jaw function, Umeå University, Umeå, Sweden

**Keywords:** Sámi health, Indigenous, ethnicity, Sweden, Sápmi, population-based, SDG 10: Reduced inequalities, SDG 3: Good health and well-being

## Abstract

**Objectives:**

Despite increasing research interest in the health and well-being of the Indigenous Sámi people, knowledge remains fragmented and insufficient. The HALDI study aims to examine health status, well-being, and associated determinants within a multi-ethnic context, focusing on the Sámi people in Sweden. This protocol outlines the study’s objectives, design, and methodology, with the goal of generating representative data to inform future research, policy, and interventions targeting Sámi health.

**Design:**

To ensure relevance, initial focus groups discussions identified key health issues for the Sámi people in Swedish Sápmi. Based on these findings, a comprehensive questionnaire was developed, encompassing socioeconomic status, ethnicity, psychiatric and somatic disorders, and self-perceived health. All adults in Jokkmokk municipality (*n* = 4077) were invited to participate. The following year, a clinical examination was conducted, including measures such as blood pressure, pulse, height, weight, blood sampling, and an updatet questionnaire on health status, dietary habits, and, for those aged 65 years and above, assessments of frailty and cognition. A total of 1682 individuals (41%) responded to the first questionnaire; 68% identified as Swedish, 22% as Sámi, and 7% with other ethnic identities. In the clinical study, 706 participated with similar ethnic proportions.

**Conclusion:**

This is the first study in Sweden to incorporate ethnic self-identification in a multi-ethnic population. By presenting the study protocol and sample characteristics, we highlight the potential for future analyses to inform evidence-based health interventions and policy to improve outcomes for the multi-ethnic Arctic population.

## Introduction

The health status of the Sámi, the Indigenous people of Norway, Sweden, Finland, and the Russian Kola Peninsula, known as the traditional Sámi region Sápmi, has garnered increasing interest in the Nordic countries. Research indicates that the Sámi face challenges in obtaining a comprehensive, updated overview of their health situation mainly due to the lack of official statistics that include ethnicity [1,2]. Sweden being a welfare state with a strong focus on health care and equality, maintains the highest standards in public health and health statistics. At the same time, Sweden lacks systematic data, impeding the understanding of the health and social well-being of its sole indigenous population - the Sámi people. This absence of robust and relevant data has prompted international criticism of Sweden [3–5]. Sámi health studies in Sweden typically rely on self-reported data through questionnaires and utilize registers such as the electoral roll of the Sámi Parliament [6], the labor statistics based on administrative sources to identify reindeer herding, and the reindeer mark register [7,8] to identify eligible study participants. However, this approach has faced criticism as these registers serve different purposes, such as the Sámi electoral roll exclusively intended for exercising parliamentary rights rather than participation in research. Internationally, United Nations (UN) and World Health Organization (WHO) highlight the importance of and fundamental right to self-identification for Indigenous Peoples and research should abide these principles [9].

Previous epidemiological studies regarding Sámi health in Sweden, based on registries for inclusion, were carried out on data from 1960 to 2008 [6]. These earlier studies reported somewhat higher standardized mortality ratios for vascular and respiratory disease in Sámi women, and for externally caused injuries leading to death in Sámi men but lower mortality in cancer among Sámi compared to the reference population [10]. The same data set also found a lower risk of overall cancer in Sámi men, but not in Sámi women, compared to a reference population [11]. A lower incidence of cancer has been found in the Sámi compared to Swedes, even though an increased risk of ovarian and gastric cancer has been observed in Sámi women, and of stomach cancer in Sámi men [7]. The observed differences in risk for cancer, as well as small differences for cardiovascular disease between Sámi and Swedish study participants, have been proposed to be associated with lifestyle-related factors such as a high-fat and high-protein diet, high levels of physical activity, and differences in alcohol and tobacco use [7]. However, these associations are inadequately studied at present.

Recent questionnaire study results of self-reported health showed poorer dental health, a higher prevalence of asthma, and overweight among the Sámi compared to the corresponding results for the reference population in Sweden. Further, the mental health status was similar or better for Sámi study participants [8]. The latter contradicts previous studies indicating more depression and anxiety among Sámi reindeer herders [12] and may be explained by differences in the study samples and methods. Thus, the current knowledge on Sámi health in Sweden is still both fragmented and outdated [1,2].

In northern Norway, a population-based study of health and living conditions, the SAMINOR study, has contributed to our knowledge of the Sámi people′s health situation in Norway since the beginning of the 2000s [13,14]. Unique to the SAMINOR study is the use of self-identification of ethnicity. The SAMINOR protocol contains both a questionnaire on the health status, and a health examination and it has been conducted twice, first during 2003–04 (13) and later during 2012–14 (14) in several municipalities within the traditional Sámi area of Norway. Currently, the third data collection in the SAMINOR 3 study is ongoing [15]. Data from the clinical SAMINOR studies indicate that the Sámi population reports poorer health compared to the general population. This includes results such as a higher prevalence of obesity in Sámi women compared to Norwegian women [16], an increased prevalence of cardiovascular disease among Sámi individuals in Norwegian-dominated areas compared to those Sámi-majority areas [17], a higher occurrence of angina pectoris in Sámi individuals relative to non- Sámi [18], and a higher age-standardized prevalence of pre-diabetes and type 2 diabetes mellitus in Sámi compared to non-Sámi individuals [19]. However, these results cannot be directly extrapolated to Swedish conditions because living conditions may vary due to differences in socio-economic situations, societal approaches to the Sámi population, and the organization of health care.

The HALDI (Health And Living conDItions in Sápmi) study initiated its collaboration with the municipality of Jokkmokk in 2015. Jokkmokk is located in the inland regions of Arctic Sweden and is part of Sápmi. Jokkmokk has been renowned for its significant Sámi presence for centuries, and until the mid-1800s the Sámi population constituted the majority in the region [20]. The HALDI study started in 2018 and is the first health study based on ethnic (indigenous) self-identification in Sweden. The HALDI study was conducted in three steps: focus group discussions with Sámi representatives in 2018–2019, a postal/digital questionnaire in 2021, and a clinical health examination alongside a repeated questionnaire in 2022. The primary objective of this paper is to provide an overview of the HALDI study, including its aims, design, participation recruitment, and the characteristics and representativeness of the participants.

The study aims to elucidate the relationship between living conditions on health and well-being within a multi-ethnic environment, encompassing the Sámi people. The study employs a multi-faceted approach to provide a comprehensive understanding of the determinants of health in an Arctic municipality. Both subjective and objective assessments of health and living conditions are applied, enabling examination of the complex interplay between environmental, social, and individual factors that contribute to health outcomes.

## Materials and methods

### Focus groups

With support from the Swedish government through the Ministry of Health and Social Affairs, the first of the three steps was to conduct focus groups in Sápmi, the traditional Sámi area of Sweden. During the autumn of 2018 and the spring of 2019, eleven focus groups discussions - each consisting of four to ten Sámi individuals diverse in age, geographical location, and occupation - were carried out in the counties of Norrbotten, Västerbotten, and Jämtland-Härjedalen in northern Sweden. The objective was to explore Sámi people’s experiences and views on health, health care and health research to ensure that their specific priorities and areas of interest were included in the upcoming development of the questionnaire. The study population was recruited through self-selection and snowball sampling methods. In total, 51 individuals took part in the discussions, of whom 66% were women. Participants ranged in age from 23 to 77 years, with a mean age of 53 years. A pre-tested interview guide with semi-structured questions was used, focusing on two overarching themes: Sámi health, and health research with an emphasis on Sámi health. The discussions were audio-recorded and conducted primarily in Swedish. When Sámi languages were spoken, they were translated and transcribed into Swedish. The data were analyzed using inductive qualitative content analysis [21].

By including members of Sámi communities, the study aimed to align its focus with relevance to their perspectives and needs. Findings from the focus groups, as well as the SAMINOR study [13], highlighting the importance of self-identification to establish ethnicity. Consequently, this criterion was incorporated into the questionnaire.

By allowing individuals to define their own ethnic identity, the study aimed to capture a more nuanced and accurate representation of the diverse backgrounds within the Sámi population [22]. Other main findings were that participants described a holistic view of health. For achieving good health, they emphasized time in nature, physical activity, and spirituality that was linked to nature rather than religion. The cultural norm ‘ieš birget’ (self-reliance) affected the willingness to seek care, promoting self-sufficiency and avoiding burdening others. Discussing mental health and substance abuse openly was seen as rare and difficult, negatively impacting health and care-seeking behavior.

Participants also reported negative experiences with healthcare due to a lack of cultural understanding and knowledge about Sámi life. Discrimination, racism, and ignorance about Sámi history and conditions were common, posing health risks. There was general skepticism towards research, stemming from a lack of involvement, poor information, and no feedback, as well as historical issues connected to racial biology. Despite this, participants recognized the need for more knowledge about Sámi health and the importance of participating in research, and they emphasized the need to clarify the purpose and benefits of research [22].

### Setting

To ensure a representative study sample that adequately included the Sámi population, Jokkmokk municipality was selected as the study site. Located north of the Arctic circle in Sweden and within the traditional Sámi area, Jokkmokk has a significant Sámi presence and serves as an important center for Sámi culture, traditions and representation. Notably, approximately 15% of its residents are registered as eligible voters in the Sámi parliament - the highest proportion of Sámi parliament voters in Sweden - underscoring the municipality’s relevance for inclusive and representative research. In 2021, a resource unit for Sámi health was established at Jokkmokk Health Center. This unit aims to enhance healthcare for Sámi patients by offering health and medical services that are culturally tailored to their needs.

### Study questionnaires

The originally planned study closely resembled the approach taken in the SAMINOR study, which involved a postal questionnaire followed shortly by a clinical examination [23]. However, due to the COVID-19 pandemic, health and safety considerations necessitated adjustments to the study protocol. As a result, the study adapted by sending the initial questionnaire in February 2021, while the clinical examination was postponed for one year until 2022. Due to this time gap, we introduced a second questionnaire to update the current health status of the participants, concurrent with the clinical examination. This adjustment allowed us to gather the data while prioritizing the safety and well-being of participants during these unprecedented times.

Hence, in February 2021, a postal questionnaire was sent to every resident 18 years of age and older in the Jokkmokk municipality (*n* = 4077). Each recipient received an invitation letter along with login details for accessing a web-based questionnaire. At request of the participants, a paper version of the questionnaire was provided in three languages: Swedish, North Sámi, and Lule Sámi.

The questionnaire encompassed a wide range of topics, including various aspects such as socioeconomic factors, language, self-reported psychiatric and somatic disorders, self-perceived health, health-related conditions (EQ-5D-3L [24], mental well-being (Short Warwick-Edinburgh Mental Well-being Scale (SWEMWBS) [25]), screening tools for stress (Perceived Stress Scale (PSS-10) [26,27]), depression and anxiety (Patient Health Questionnaire-4 (*PHQ-4)* [28]), oral health including orofacial pain and jaw function [29], current medication, use of health services, exposure to violence, smoking habits, physical activity, and a food intake questionnaire. Ethnicity was determined based on self-identification, following advice from the focus group results (see below). A key finding from the qualitative focus groups was participants’ experiences of discrimination and racism. Based on this, several specific questions were developed and included in the questionnaire. Regarding experiences of historical losses, our measurement of intergenerational trauma utilizes a composite instrument conceptually informed by the Historical Loss Scale (HLS) and the Historical Trauma Response Scale (HTRS) [30]. Consistent with our community-engaged approach, the final set of questions was adapted and abbreviated based on findings from our focus groups, which identified historical loss and discrimination as key health concerns within this population. Two questions were used to assess the household economic situation, similar to those in the Swedish national public health survey [31]. The first question was: *‘Would you or your household be able to pay an unexpected expense of 12,000 kronor within a month without borrowing or asking for help?’* Possible responses were *‘Yes’, ‘No’,* and *‘Don’t know’.* The second question asked: ‘*Have you experienced difficulties in managing ongoing expenses for food, rent, bills, etc., in the past 12 months?’.* The response options were *‘Yes, on one occasion’, ‘Yes, on several occasions’, and ‘No’.*

Additionally, three questions from the Alcohol Use Disorders Identification Test – Consumption (AUDIT-C) were used to estimate alcohol use [32,33]. For more details, see Table S1.

Accordingly, the questionnaire encompassed a wide array of research topics, incorporating both previously validated items from comparable surveys and newly developed questions. A detailed discussion of each individual question is beyond the scope of this paper. However, future publications based on data from the HALDI study will provide comprehensive methodological descriptions of these elements when presenting future research findings

In total, the questionnaire comprised 112 questions and the questionnaire was open 13 weeks (22 February − 24 May 2021).

### Study participants

**Figure 1.**
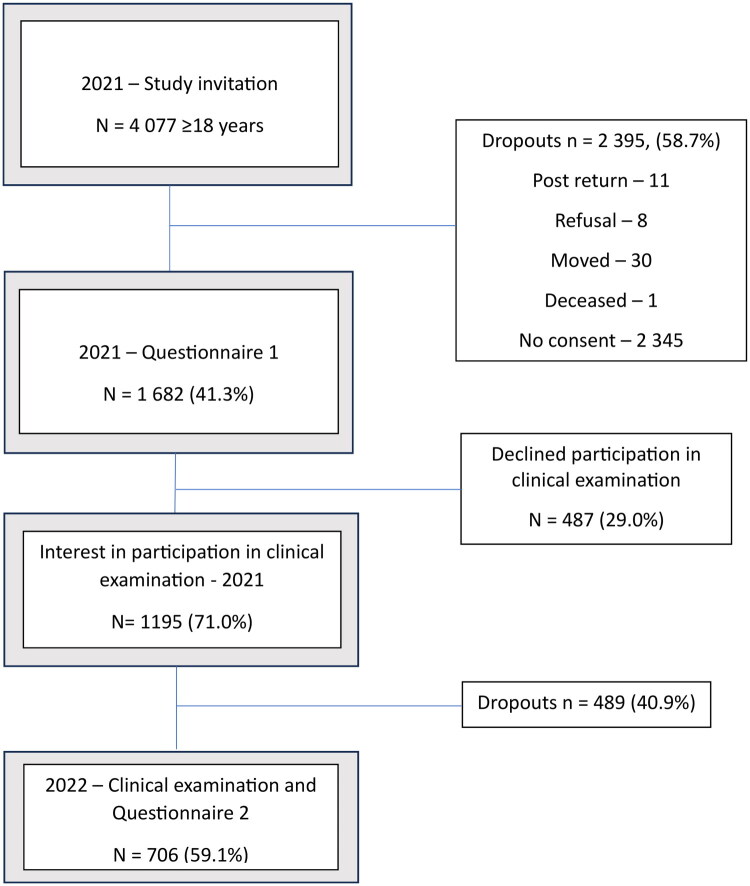
Flow chart of the study cohort.

A total of 1 682 individuals, representing 41.3% of the total invited population, responded to the questionnaire. There were 2 395 dropouts observed. Out of these, 2 370 individuals did not provide any response to the questionnaire. Thirty individuals were identified as having moved, eleven individuals either returned empty questionnaires (nine) or post returned (two), eight individuals actively declined to participate, and one individual was reported deceased after the initial mailing (see Figure 1). Out of the study participants, 757 (45.0%) responded using the paper questionnaire, while 925 (55.0%) opted for the web-based questionnaire. None of the respondents requested the questionnaire in Sámi language. Further information on the study participants is presented in Table 1.

**Table 1.** Background characteristics of the study participants in questionnaire 1.

	Swedish		Sámi		Other ethnic identity		No ethnicity information		
	n	%	n	%	n	%	n	%	p
**Total**	1144	68.0	375	22.3	113	6.7	50	3.0	
**Sex**									<0.001^a**^
Man	505	44.1	123	32.8	50	44.2	15	30.0	
Woman	629	55.0	246	65.6	62	54.9	23	46.0	
Other/missing	10	0.9	6	1.6	1	0.9	12	24.0	
**Age, years**									<0.001**
Mean (SD)	62.1 (16.3)		51.8 (17.1)		54.6 (16.1)		59.1 (23.1)		
Min-Max	18.1–100.0		18.1–93.1		19.5–86.3		20.0–100.0		
**Age group**									
18–29 years	54	4.7	46	12.3	12	10.6	8	16.0	<0.001^a**^
30–44 years	143	12.5	90	24.0	16	14.2	10	20.0	
45–64 years	376	32.9	139	37.1	50	44.2	8	16.0	
≥65 years	571	49.9	100	26.7	35	31.0	24	48.0	
**Education**									
**Boarding school**	44	(3.9)	92	(24.9)	6	(5.4)	5	(14.3)	<0.001^a**^
*Missing data, n = 33*									
**Education level**									
≤9 years	264	23.1	44	11.8	15	13.4	14	35.9	<0.001**
10–13 years	467	40.9	118	31.6	29	25.9	15	38.5	
>13 years	355	31.1	191	51.2	60	53.6	7	17.9	
Other education	53	4.8	20	5.4	8	7.1	3	7.7	
*Missing data, n = 17*									
**Other household language**	58	5.1	179	48.8	75	68.8	10	28.6	<0.001**
*Missing data, n = 35*									
**Housing**									
Own house	848	75.2	272	73.9	74	66.7	24	64.9	<0.05^a*^
Condonium	17	1.5	13	3.5	1	0.9	0	0.0	
Rental	243	21.6	75	20.4	31	27.9	11	29.7	
Other	19	1.7	8	2.2	5	4.5	2	5.4	
*Missing data, n = 39*									
**Living alone**	265	23.2	76	20.3	25	22.1	14	33.3	0.247
*Missing data, n = 8*									
**Number of children**									
Mean, SD	2.18	2.29	2.15	1.21	1.97	1.13	2.6	1.71	0.606
**Employment, n = 846**									
Fulltime	422	76.0	143	64.7	45	67.2	3	100.0	<0.05^a*^
Part time	106	19.1	64	29.0	20	29.9	0	0.0	
**Seasonal work**	38	3.3	39	10.4	4	3.5	0	0	<0.001^a**^
**Household economy**									
Difficult paying unexpected expense within one month	190	17.2	87	23.4	30	27.0	2	13.3	<0.01^a*^
*Missing data, n = 79*									
Difficult paying ongoing expenses the past year	96	8.6	69	18.5	18	16.2	2	12.5	<0.001^a**^
*Missing data, n = 71*									
**Alcohol consumption**									
AUDIT-C – risk	221	20.0	75	20.9	11	10.2	2	10.5	<0.05^a*^
*Missing data, n= 92*									
**Smoking**									
Ever smoked	562	49.9	157	42.2	54	47.8	10	45.5	0.084
Daily smoker	82	7.3	17	4.6	7	6.2	3	13.6	0.168
*Missing data, n = 48*									

^a^Fisher Exact Test, *<0.05, **<0.001.

Data are presented based on self-identication of ethnicity (total *n* = 1 682).

### Self-reported ethnicity

Ethnicity was determined by the following question: ‘How do you identify yourself?’ with three response alternatives: ‘Swedish’, ‘Sámi’, and ‘Other, please specify’. They had the opportunity to select one or several of these options. Those who chose ‘Sámi’, either individually or in combination with other options, were categorized as belonging to the Sámi group. Those who chose ‘Other’, either individually or in combination with other options than ‘Sámi’, were categorized as belonging to the Other ethnic identity group. By this criterion, a total of 1144 (68.0%) was identified as Swedish, 375 (22.3%) as Sámi, and 113 (6.7%) as Other ethnic identity. Fifty cases (3.0%) refrained from indicating their ethnicity (Table 1).

### Clinical examination

The first questionnaire included the option to indicate interest in participating in the clinical examination. Of the 1682 questionnaire participants, 1195 (71.0%) expressed an interest in the clinical examination, and they were all invited through a scheduled appointment one year later, with the option to reschedule. Upon arrival at the study site, all participants completed a questionnaire with updated information regarding their health status to obtain an up-to-date overview prior to the clinical examination. This second questionnaire included most of the questions and scales from the first questionnaire administered in 2021 and also incorporated several additional items addressing the effects of the Covid-19 pandemic. For more details, see Table S2. A food and drink frequency questionnaire (FFQ2020, designed within the Northern Sweden Diet Database) was used to capture dietary habits over the past year [34].

The following measurements were included in the clinical investigation: systolic and diastolic blood pressure and pulse recordings, body height, weight, and waist and hip circumference. Blood pressure was measured using an Omron M3 automatic device. The patient was seated with the right arm resting on a table. Blood pressure and pulse were measured three times at one-minute intervals. The first reading was discarded, and the mean systolic and diastolic blood pressure, as well as pulse rate, were calculated from the second and third measurements. Body weight was measured using a Lidén Weighing Floor Scale, Model MBPA-250, with participants wearing light clothing and no shoes, and was recorded in kilograms to one decimal place. Height was measured without shoes in centimeters to one decimal place. Body mass index (BMI) was calculated as weight in kilograms divided by height in meters squared (kg/m^2^). Waist circumference was measured midway between the lowest rib and the iliac crest, with the participant standing and wearing light clothing. Hip circumference was measured at the point over the buttocks corresponding to the largest circumference.

Non-fasting blood samples were collected and transported each afternoon to the Laboratory of Clinical Chemistry, Sunderby Hospital for analysis of blood status, HbA1C, CRP, Apolipoprotein A1 and B, total cholesterol, HDL- and LDL-cholesterol, iron, transferrin, ferritin, folate, vitamin B12, calcium ion, and cystatin C levels. For laboratory methods, see Table S3.

Study participants aged 65 years and older also underwent cognitive testing with the Montreal Cognitive Assessment (MoCA) to assess overall cognitive function [35], the Symbol Digit Substitution Test [36] to assess executive function, cognitive speed, and working memory, the Trailmaking A and B tests [37] to assess processing speed, symbol recognition, and the ability to maintain two thought processes simultaneously. Finally, basic reading skills were measured using a Swedish version of the Test of Word Reading Efficiency [38] measuring word decoding and word recognition.

Additionally, muscular strength in lower extremities was measured using the chair-stand test [39], in which the participant was seated on a chair without armrests, positioned against a wall with their back in contact with the chair and feet flat on the floor. With arms crossed, participants were instructed to stand up and sit down without using their arms as many times as possible within 30 s. The number of times the participant stood up fully was recorded. Muscular strength in the upper extremities was measured by assessing hand grip strength using a Jamar hydraulic hand dynamometer (FS658). The dynamometer was adjusted to fit the participant’s hand. The participant was seated at a table with the elbow resting on the table and the forearm parallel to the tabletop. Peak strength was measured by having the participant squeeze the dynamometer with maximal effort. The test was performed three times with each hand, and the results were recorded in kilograms. For statistical analyses, the maximum value will be used.

Finally, these elderly participants also completed the Tilburg Frailty Indicator (TFI), a screening instrument for frailty [40,41].

After the examination, all participants received written information about the clinical measurements, such as blood pressure, pulse, height, weight, and waist and hip measurements. The lab results were reported within a week, and in the event of abnormalities, the study participants were notified by letter. Cases that were assessed to have a medical need for faster follow-up - either due to abnormal blood pressure or pulse measurements, or abnormal lab results - were referred to the nearby healthcare center by the two medical doctors participating in the project. In the event of a more serious abnormality, an urgent referral was made.

The clinical examination was delayed one year due to the Covid-19 pandemic and performed over eight weeks, from 15th February to 8th April 2022 and a total of 706 individuals (59.1%) participated. Of those, 467 (66.1%) identified as Swedish, 179 (25.4%) as Sámi, 50 (7.1%) as Other, whereas 10 (1.4%) refrained from indicating their ethnicity. Further information about the clinical examination participants is presented in Table 2.

**Table 2.** Characteristics of the study participants in the clinical study.

	Swedish		Sámi		Other ethnic identity		No ethnicity information		
	n	%	n	%	n	%	n	%	p
**Total**	467	66.1	179	25.4	50	7.1	10	1.4	
**Sex**									<0.001^a**^
Man	188	40.3	50	27.9	17	34.0	3	30.0	
Woman	277	59.3	127	70.9	32	64.0	5	50.0	
Other/missing	2	0.4	2	1.1	1	2.0	2	20.0	
**Age, years**									<0.001**
Mean (SD)	65.6 (13.3)		54.8 (16.4)		59.3 (13.3)		68.2 (16.2)		
Min–Max	25.2–92.7		19.0–89.3		27.9–82.2		30.2–84.8		
**Age group**									
18–29 years	6	1.3	14	7.8	2	4.0	0	0.0	<0.001^a**^
30–44 years	40	8.6	46	25.7	5	10.0	1	10.0	
45–64 years	161	34.5	64	35.8	25	50.0	3	30.0	
≥65 years	260	55.7	55	30.7	18	36.0	6	60.0	
**Education**									
**Boarding school**	16	3.5	49	27.8	2	4.0	2	28.6	<0.001^a**^
*Missing data, n = 11*									
**Education level**									
≤9 years	76	16.3	17	9.5	5	10.0	3	30.0	<0.001^a**^
10–13 years	218	46.7	52	29.1	15	30.0	6	60.0	
>13 years	173	37.0	110	61.5	30	60.0	1	10.0	
*Missing data, n = 0*									
**Other household language**	20	4.3	106	59.9	30	61.2	2	33.3	<0.001^a**^
*Missing data, n = 11*									
**Housing**									
Own house	369	79.7	148	83.6	37	74.0	6	85.7	0.639
Condonium	5	1.1	1	0.6	0	0.0	0	0.0	
Rental	83	17.9	25	14.1	11	22.0	1	14.3	
Other	6	1.3	3	1.7	2	4.0	0	0.0	
*Missing data, n = 9*									
**Living alone**	103	22.1	35	19.6	11	22.0	2	25.0	0.860
*Missing data, n = 2*									
**Number of children, n= 570**									
Mean, SD	2.16	1.04	2.14	1.12	2.15	1.06	2.71	2.43	0.603
**Employment, n = 349**									
Full time	158	75.2	67	60.9	21	77.8	2	100.0	0.114
Part time	42	20.0	37	33.6	6	22.2	0	0.0	
**Seasonal work**	14	3.0	19	10.6	1	2.0	0	0.0	<0.001^a**^
**Household economy**									
Difficult paying unexpected expense within one month	66	14.4	4 1	23.0	9	18.0	0	0.0	0.064
*Missing data, n = 15*									
Difficult paying ongoing expenses the past year	31	6.8	32	18.0	6	12.0	0	0.0	<0.001^a**^
*Missing data, n = 16*									
**Alcohol consumption**									
AUDIT-C – risk	85	18.6	27	15.8	3	6.5	0	0.0	0.124
*Missing data, n = 25*									
**Smoking**									
Ever smoked	226	48.8	70	39.5	26	52.0	4	57.1	0.140
Daily smoker	30	6.5	8	4.5	3	6.0	1	14.3	0.430
*Missing data, n = 9*									

^a^Fisher Exact Test, *<0.05, **<0.001.

Data are presented based on self-identification of ethnicity (total *n* = 706).

### Data management

In accordance with university guidelines, the HALDI study has established a comprehensive Data Management Plan that meticulously addresses all stages of data handling, from collection to archiving. The study employs rigorous protocols for data collection, quality assurance, and secure storage, incorporating information classification as well as risk and vulnerability analyses in line with Umeå University’s requirements. These measures are designed to safeguard participant confidentiality while ensuring data integrity and reliability. Such robust data management is particularly critical given the sensitive nature of the personal data involved in the HALDI study.

### Statistics

Statistical analyses were conducted using IBM SPSS Statistics Version 29.0. Categorical variables are presented as numbers and percentages, and group differences are analyzed with the chi-squared test. The continuous variables are presented as means and standard deviations (SD), and one-way ANOVA was used to detect groups differences.

Educational level was categorized based on the highest reported level of education into the following groups: ≤9 years, 10–13 years, >13 years and other education. Economic capacity was assessed through participants’ reports of ability in paying an unexpected expense within one month, with responses dichotomized as Yes (‘Yes’) or No (‘No’ or ‘Don’t know’), or difficulties in managing ongoing expenses over the past year, with response dichotomized as Yes (‘Yes, at one occasion’ or ‘Yes, on several occasions’) and No (‘No’).

To estimate alcohol use, participants were classified as either AUDIT-C risk users or non-risk users based on their scores and gender-specific cutoff values (refer to the previous section).

To investigate independent associations between variables of interest and various health outcomes, regression analyses will be used. When relevant, mediation analysis will be used to explore the mechanisms or pathways underlying observed effects.

## Ethics and dissemination

The study was conducted in accordance with the Declaration of Helsinki [42], furthermore the Ethical Guidelines for Sámi health Research and Research on Sámi Biological Material were followed [43]. The study obtained collective consent from the Sámi Parliament in Sweden (Dnr 1.2.6-2016-1530). All participants received information about the study and gave written consent to participate. Ethical approval was given for all parts of the study by the Swedish Ethical Review Authority. The focus group study was approved by the Regional Ethical Review Board of Umeå (Dnr 2017/408-31). Both the questionnaire study and the clinical examination were approved by the Ethical Review Authority of Stockholm (Dnr 2020-03622 and Dnr 2021-05837-01, 2022-00699-02 respectively).

## Baseline characteristics of study participants

The main purpose of this paper is to describe the study design, including participant recruitment, its importance, and to prepare for upcoming papers detailing research questions addressing the interplay of ethnicity, socioeconomic aspects, living conditions, and perceived psychical and mental health in an Artic population. Therefore, in this section, we will only provide some basic descriptive findings to characterize and illustrate the representativeness of the study participants.

### Questionnaire 2021

The baseline characteristics are presented in Table 1. The Sámi group had a significantly higher proportion of women (65.6%) compared to the other groups. Both the Sámi and Other ethnic identity groups were younger, with mean (SD) ages of 51.8 (17.1) and 54.6 (16.1) years, respectively, compared to Swedish and those with no ethnicity information. These age differences were also reflected in a higher percentage of Sámi participants in the younger age groups and a lower percentage in the 65 years and older category. Additionally, a greater percentage of Sámi participants reported having attended boarding school (24.9%). While Sámi study participants had a higher educational level than the Swedish group, the Other ethnic identity group reported a higher education level than both the Sámi and the Swedish groups. Furthermore, another household language was spoken in 68.8% of the Other ethnic identity group, 48.8% of the Sámi group, and in 28.6% of the group with no ethnic information. All of these were significantly higher than in the Swedish group, where only 5.1% reported speaking another language at home or in the daily life.

A lower proportion of the Other ethnic identity group lived in their own house (66.7%) compared to the Swedish (75.2%) and Sámi groups (73.9%). There were no significant differences in the proportion of individuals living alone or in the number of children among the groups. Regarding employment, the Sámi group had a lower rate of full-time employment and a higher proportion of seasonal work.

Both the Sámi and Other ethnic identity groups reported a lower household economic capacity regarding their inability to pay an unexpected expense within one month, with 23.4% and 27.0%, respectively, compared to 17.2% in the Swedish group. They also reported difficulties in managing ongoing expenses over the past year, with 18.5% and 16.2%, respectively, compared to 8.6% in the Swedish group. The percentages of AUDIT-C risk users were statistically higher in the Swedish (20.0%) and Sámi (20.9%) groups compared to the Other ethnic identity (10.2%) and No ethnicity groups (10.5%); however, there was no significant difference between the Swedish and Sámi participants. Additionally, there were no differences in reported smoking habits among the groups. For further details, see Table 1.

### Clinical examination 2022

The baseline characteristics of the study participants in the clinical examination are presented in Table 2. The Sámi group had a significantly higher proportion of women (70.9%) compared to all other groups and they were significantly younger than the Swedish and no ethnicity groups. Both the Sámi and Other ethnic identity groups also had a lower percentage of study participants 65 years and older. The Sámi participants reported higher attendance at boarding school. Both the Sámi and Other ethnic identity groups had significantly higher education levels compared to the Swedish group. Another household language was spoken in a higher percentage in the Sámi and Other ethnic identity groups compared to the Swedish. There were no differences in housing, living alone, or the number of children between the groups. However, the Sámi group had a higher percentage of seasonal work. Regarding household economy, the Sámi reported a lower capacity to handle both unexpected expenses within one month and ongoing expenses over the past year compared to the Swedish and Other ethnic identity groups. There were no differences in alcohol risk use or reported smoking habits between the groups. For further details, see Table 2.

## Discussion

The HALDI study is the first research initiative in Sweden to employ ethnic self-identification for examining health and living conditions within a multi-ethnic population, including the Sámi people from the municipality of Jokkmokk in Sweden. The overall aim of the study was to investigate differences in living conditions and the health status between those identifying as Sámi and the majority population of Swedes living in the same municipality. The primary objective of this article was to present the aims, design and participation of the study.

One main issue addressed in the focus group discussions by our informants was how to best capture Sámi identity. It became evident that ethnic self-identification was fundamental, rather than being classified by registries primarily used for participating in a free voting democracy. Within the focus groups, there was a clear consensus that inquiries regarding proficiency in the Sámi languages as a means to capture ethnicity were not appropriate. Such an approach was considered to risk reinforcing a sense of exclusion, given that many Sámi individuals lack proficiency in their native Sámi languages [22]. Furthermore, our focus groups revealed that perceived discrimination and racism are key aspects of health for the studied population. Therefore, we included several questions on this topic in the questionnaire (See Supplement). In order to create a solid foundation for our forthcoming work we have recently published a scoping review on methods, data and definitions in research on racism and ethnic discrimination in arctic populations [44].

Equally important, these focus groups contributed to our understanding of medical and life conditions important for Sámi health. All these aspects have been incorporated into our questionnaires and clinical examination. This first step in the project was crucial for ensuring Sámi participation in the planning and execution of the study. Important areas were highlighted that have traditionally not been included in studies concerning Sámi health. For example, spirituality was noted as an important factor for maintaining good health, along with an emphasis on addressing more sensitive issues such as mental health and alcohol use [22].

The choice of study location was based on the presence of a sufficiently large proportion of Sámi residents to ensure a representative study group. Since there are no ethnic registries in Sweden, we selected an area with the highest percentage of registered voters in the Sámi Parliament. Our data consisted of 22% of participants who self-identified as Sámi, which is considered reasonable, although it may constitute an underrepresentation of the Sámi group. However, we cannot investigate this further due to the lack of available data, as previously described.

Overall, the response rate for the first questionnaire was 41%, which is in line with other population questionnaire studies [45,46]. In fact, concurrently with the HALDI questionnaire, two other study surveys were distributed during approximately the same time period to parts of the same area and population [45], which may have negatively affected the response rate for all the studies. The study’s establishment within both the Sámi Parliament and Sámi civil society, facilitated by Sámi participation and influence in the focus groups, does not appear to have resulted in an increased rate of study participation. Similarly, the availability of the option to complete the questionnaire in two different Sámi languages did not seem to influence the participation, as none of the study participants chose to use this option. However, it is still possible that our dataset is biased toward non-Sámi-speaking Sámi individuals, as none of our respondents answered the survey in Sámi.

Our statistical analysis indicated significant differences in several socioeconomic factors between the Swedish and Sámi study participants. The Sámi participants were predominantly women and were more highly educated, consistent with recent questionnaire findings [45]. However, they were younger compared to the previous study, which highlights differences in the study samples. Furthermore, the Sámi had less full-time employment and a higher proportion of seasonal work, as well as a weaker household economy compared to the Swedish participants. There were no differences in alcohol or tobacco use between the Sámi and Swedish groups, but both groups consumed more alcohol than the Other ethnic identity group.

The participation in the clinical examination one year later, in 2022, consisted of 59% of those who had indicated an interest in participating in the initial questionnaire. Consequently, 12%, or just over 200 study participants, were lost during that year of the COVID-19 pandemic, which is unfortunate but understandable because of the recommendations for social distancing and a fear of engaging in public activities [47]. For study participation, we observed a remaining bias in the study population, with a discrepancy characterized by a higher proportion of women, younger ages, higher education, and poorer household economy in the Sámi group compared to the Swedish group.

Since our assumption is that the Sámi portion of the population is not predominantly composed of younger, more highly educated women, but rather that this observation results from the underrepresentation of older men and individuals with lower educational attainment in the study, we will need to adjust for these specific parameters in upcoming analyses concerning various planned health outcomes. There are several options for doing this. The first, and perhaps most common, is to use age, gender, and, where applicable, educational level as covariates in the statistical analyses. This approach is uncontroversial and widely applied. Another option is to weight the data for gender and age based on overall population statistics. Such data are readily available for each municipality and year through Statistics Sweden, making this a viable alternative as well. Hence, we believe that with an understanding of the bias in the study population, there are significant opportunities to contribute knowledge about living conditions and health outcomes in the various groups within the studied area. However, we acknowledge that there may still be some uncertainties in the coming results, which highlight the need for more comprehensive data collection in the future to adequately describe the area.

## Conclusion

The HALDI study employs a multi-faceted approach that utilizes both subjective and objective assessments of health and living conditions. It is the first research initiative to incorporate ethnic self-identification within a multi-ethnic population, including the Sámi people in northern Sweden. In this paper, we present the study protocol and discuss the representativeness of the study sample, which was likely compromised due to the COVID-19 pandemic. The study was initiated with focus groups that provided input on areas important for describing the concept of Sámi health. Furthermore, we conducted a questionnaire that could be answered online or on paper, and in the following year, we invited participants to a clinical examination that included measurements of vital signs such as pulse and blood pressure as well as blood sampling and a panel of assessments specifically targeting older individuals, including frailty assessments.

We posit that forthcoming analyses could significantly advance our understanding of health conditions and inform the development and implementation of evidence-based interventions and policies designed to improve health outcomes for the multi-ethnic population residing in the Arctic municipality. Disseminating results to the participating municipality will assist local, regional, and national policymakers in formulating effective health initiatives to enhance healthcare delivery in the future. Ultimately, this will benefit individuals, the Sámi community, and Swedish society at large.

## Supplementary Material

Supplementary Table S1.docx

Supplementary Table S2.docx

Supplementary Table S3.docx

## Data Availability

Due to the sensitive nature of the data including confidential health information and ethnic identifiers, the data underpinning the findings presented in this article are not publicly accessible. Further inquiries regarding data availability should be directed to the corresponding author.
