# Supplementary material for: Protocol for the HALDI study—conceptual framework for investigating health and living conditions in an arctic area of Sweden with a multiethnic population
Source: Ann Med. 2025 Jul 28;57(1):2537914. doi: 10.1080/07853890.2025.2537914 (PMC12308873; doi:10.1080/07853890.2025.2537914)
Supplement: Supplementary Table S1.docx [file IANN_A_2537914_SM0815.docx]

Supplementary Table S1. Questionnaire HALDI study 2021

| **Question** | **Reply Options** |
| --- | --- |
| 1. I consent to participate in the survey in accordance with the written information I have received in the accompanying research subject information. | Yes / No |
| 2. I am interested in being contacted for a clinical health examination planned at a later date within HALDI. I am aware that this is only an expression of interest and not in any way binding. | Yes / No |
| 3. I consent to my survey responses being linked with national register data such as Statitistics Swedens’s business register, the pharmaceutical register, and the national patient register. | Yes / No |
| 4. What year were you born? | Year (YYYY) |
| 5. How do you define your gender identity? | Man / Woman / Other / Prefer not to say |
| 6. How many people live in your household? | Number of people |
| 7. Who do you live with? (Who you live with at least half the time.) | No one (live alone) / Partner (living separately) / Parents or siblings / Spouse/partner/cohabitant / Other adults / Children |
| 8. If you have children: how many? | Number of children |
| 9. How do you live? | Own house/terraced house / Condominium/co‑operative apartment / Rental apartment / Lodger/student apartment /room / Service apartment/sheltered housing / Nursing home/ Senior housing / Other |
| 10. What is your highest completed education? | Primary school/elementary school/lower secondary school or similar / 2‑year high school or vocational school / 3–4 year high school / Folk high school or similar / University/college < 3 years / University/college ≥ 3 years / Other education |
| 11. Did you live in a boarding-/nomad school when you attended primary school? | Yes / No |
| 12. Do you speak any language other than Swedish at home/daily? | Yes (which: ____) / No |
| 13. How would you assess your general state of health? | Very good / Good / Neither good nor bad / Bad / Very bad |
| 14. How tall are you (in full cm)? | cm (numeric entry) |
| 15. How much do you weigh (in full kg)? If you are pregnant, please state your usual weight | kg (numeric entry) |
| 16. Have you ever been diagnosed by a doctor with any of these medical conditions? (Check all that apply.) | Type 1 Diabetes / Type 2 Diabetes / Hypertension / Hyperlipidemia / Myocardial Infarction / Angina Pectoris / Heart Failure or Other Cardiac Disease / Stroke / Asthma / Allergies Requiring Treatment / Chronic Bronchitis, Emphysema, COPD / Rheumatic Disease / Fibro-myalgia / Chronic Pain / Osteoporosis / Osteoarthritis / Metabolic Disorders / Parkinson’s Disease / Multiple Sclerosis (MS) / Dementia or Memory Disorder / Gastrointestinal Disease / Cancer / Sleep Apnea / Gluten Intolerance / Lactose Intolerance / Milk Allergy / None of the Above |
| 17. Have you ever been diagnosed by a doctor with any of these mental health conditions? (Check all that apply.) | Depression / Anxiety/worry / Sleep disorder / Obsessive‑compulsive disorder (OCD) / Eating disorder / Other psychiatric disorder / None of the above |
| 18. Have you ever been diagnosed by a doctor with any functional impairment? (Check all that apply.) | Neuropsychiatric disability (e.g. ADHD, Autism) / Intellectual disability before age 18 / Physical disability / Other disability / None of the above |
| 19. Are you currently taking any prescription medication? | Yes (state how many: ____) / No *(If No, skip to Q21)* |
| 20. Which medication(s) do you take regularly? (Check all that apply.) | Oral antidiabetic / Insulin / Antihypertensive / Lipid-lowering / Cardiac or angina medication / Anticoagulants / Asthma or COPD medication / Corticosteroids or anti-inflammatory / Ulcer medication / Cancer treatment drugs / Metabolic disorder medications / Hormonal therapy / Analgesics / Antidepressants / Sedatives / Hypnotics / Herbal or natural remedies / Dietary supplements / Other |
| 21. For each statement, which best describes your health condition today? | Walking: No problems / Some problems / Bedridden Personal hygiene: No problems / Some problems / Can't wash/ dress self Daily activities: No problems / Some problems / Can't perform them Pain & discomfort: None / Moderate / Severe Anxiety & depression: Neither / Somewhat / Very |
| 22. Have you ever had pain in your muscles and joints lasting ≥3 months during the last year? | Yes / No *(If No, skip to Q26)* |
| 23. Where do you have or have had pain/stiffness? (See pain drawing.) | Neck / Chest / Upper back / Lower back / Thigh / Lower leg / Jaw / Shoulder/scapula / Elbow / Hip / Wrist/finger / Knee / Ankle/foot |
| 24. Do you have pain in both the left and right halves of your body? | Yes / No |
| 25. Has the pain interfered with your daily activities? | Yes, at work / Yes, in my free time / No |
| 26. Do you have pain in your temple, face, jaw, or jaw joint once a week or more often? | Yes / No |
| 27. Do you have pain opening your mouth wide or chewing once a week or more often? | Yes / No |
| 28. Does your jaw lock or become stuck once a week or more? | Yes / No |
| 29. If you answered yes to any of Q26–28, have you also sought treatment? | Yes, from a doctor / Yes, from a dentist / Yes, from another healthcare provider / Yes, from an alternative practitioner / No |
| 30. Have you experienced migraines during the last 12 months? | Yes, a few times / About once a month / About once a week / Several times a week / Daily / No, never |
| 31. Have you experienced headaches during the last 12 months? | Yes, a few times / About once a month / About once a week / Several times a week / Daily / No, never |
| 32. Has your general state of health been negatively affected by the Covid‑19 pandemic? | Yes / No |
| 33. Indicate how well the following statements correspond to your experience over the last 2 weeks. | [For each:] Always / Most of the time / Sometimes / Rarely / Never: • I have had a positive view of the future • I have felt that I have been useful • I have felt calm • I have handled problems well • I have thought clearly • I have felt close to other people • I have been able to decide things for myself |
| 34. Over the past 2 weeks, how often have you been bothered by the following? | Not at all / Several days / More than half the days / Almost every day: • Little interest or pleasure • Feeling down, depressed, or hopeless • Feeling nervous, anxious, or very stressed • Unable to stop or control worrying |
| 35. If you checked any problem in Q34, how much difficulty has it caused in work/home/relationships? | No difficulty / Some difficulty / Great difficulty / Extreme difficulty |
| 36. During the past month, how often have you felt/thought the following? | Never / Quite rarely / Sometimes / Quite often / Very often: • Unable to control important things • Able to handle personal problems • Felt things were going your way • Felt difficulties were piling up to the point where they are unmanageable |
| 37. What in your environment is important for health and well‑being (scale 1–10)? | No importance (1) to Great importance (10): • Nature • Art, crafts, music, film, literature • Human relationships • Spirituality • Other (specify): ______ |
| 38. How do you sleep? | Very good / Good / Neither good nor bad / Bad / Very bad |
| 39. On average, how many hours of sleep do you get on a typical weekday? | Less than 6 hours / 6–10 hours / More than 10 hours |
| 40. Do you work shifts (evening or night)? | Yes / No |
| 41. Rate your physical activity (1 very low–10 very high) at: a) 14 years of age b) 30 years of age c) Today | 1 (Very low) – 10 (Very high) for each period |
| 42. Have you experienced an injury/accident requiring medical treatment (excluding violent crime)? | Yes, in the last 12 months / Yes, more than 12 months ago / No, skip to Q46 |
| 43. If yes to Q42, what type/location of accident? (Check all that apply.) | At work / At home / In leisure time:   - Car - Motorcycle - Snowmobile - ATV - Tractor - Fall accident - Cut injury - Other |
| 44. Has the accident(s) resulted in reduced working capacity? | Completely / Partially / Not at all |
| 45. Has the accident(s) resulted in reduced physical activity in your leisure time? | Completely / Partially / Not at all |
| 46. Are you born in Sweden? | Yes *(If Yes, go to Q49)* / No |
| 47. If no, what country were you born in? | Country (text entry) |
| 48. What year did you move to Sweden? | Year (YYYY) |
| 49. How do you identify yourself? (Check all that apply.) | Swedish / Sami / Other (specify): ____ |
| 50. Do you have negative experiences/memories regarding yourself or someone in your family that affect you today? | Yes / No |
| 51. If yes to Q50, how often do you think about the following? | Every day/Every week/Every month/Annually or special occasions/Never: • Loss of land • Loss of language • Loss of family ties due to boarding school/fleeing/forced relocation • Loss of culture/lifestyle • Loss of religion • Other (specify): ____ |
| 52. What have been your main sources of income in the last year? (Check all that apply.) | Full-time / Part-time / Seasonal / Salaried employment / Self-employed / Old‑age pension / Parental benefit / Sickness benefit / Sickness compensation / Unemployment benefit / Social assistance / Support from family / Loans/student loans/scholarships / Other (savings/inheritance, etc.) |
| 53. If self‑employed, what industry do you work in? | Administration/economy / Fishing / Agriculture / Culture / Reindeer husbandry / Forestry / Other (specify): ____ |
| 54. How often or always do the following describe your work? | Very often/always;Quite often;Sometimes;Quite rarely;Very rarely/never: • Work physically demanding • Work very sedentary • Need to lift heavy objects • Repetitive / monotonous movements • Exposed to strong shaking/vibrations • Work mentally demanding • Time for recovery • Experience stress |
| 55. Overall, how satisfied are you with your current job? | Very satisfied / Satisfied / Neither satisfied nor dissatisfied / Dissatisfied / Very dissatisfied |
| 56. Could you or your household pay SEK 12 000 within a month without borrowing/help? | Yes / No / Don’t know |
| 57. In the last 12 months, have you had difficulties managing ongoing expenses? | Yes, once / Yes, several occasions / No |
| 58. How has your economy been affected by the Covid‑19 pandemic? | Improved / No change / Deteriorated |
| 59. Can you get help from people if you have practical problems or are sick? | Always / Most of the time / Rarely / No, never |
| 60. Do you have one or more people you can confide in? | Always / Most of the time / Rarely / No, never |
| 61. Have you lost someone close to you through suicide? | Yes / No |
| 62. Have you ever seriously considered taking your own life? | Yes, >12 months ago / Yes, in last 12 months / No, never |
| 63. Have you ever attempted suicide? | Yes, >12 months ago / Yes, in last 12 months / No, never |
| 64. If yes to Q63, did you tell anyone about it? (Check all that apply.) | Family / Friends / Professionals / No |
| 65. Do you smoke or have you previously smoked? | Yes, daily / Yes, occasionally / Yes, previously / No, never |
| 66. Do you use or have you used snus? | Yes, daily / Yes, occasionally / Yes, previously / No, never |
| 67. How often do you drink alcohol (last year)? | Monthly or less / 2–4×/month / 2–3×/week / ≥4×/week / Never *(If Never, skip to Q70)* |
| 68. How many glasses do you drink on a typical day when you drink alcohol? | 1–2 / 3–4 / 5–6 / 7–9 / 10 or more |
| 69. How often do you drink six or more glasses on the same occasion? | Daily/almost daily / Weekly / Monthly / Less often than monthly / Never |
| 70. Do you or have you had periodic heavy alcohol consumption? | Yes, in last 12 months / Yes, previously / No |
| 71. Have you ever used illicit drugs? | Yes, in last 12 months / Yes, previously / No: • Hash/marijuana • Other drugs |
| 72. Have you experienced discrimination in the last 2 years? | Very rarely/never / Quite rarely / Sometimes / Quite often / Very often or always |
| 73. Have you previously experienced discrimination? | Very rarely/never / Quite rarely / Sometimes / Quite often / Very often or always |
| 74. Why do you think you were discriminated against? (Check all that apply.) | Ethnicity / Disability / Geographical affiliation / Learning difficulties / Sex / Transgender identity or expression / Nationality / Religion or belief / Sexual orientation / Illness / Don’t know / Other |
| 75. Where did the discrimination take place? (Check all that apply.) | Internet / School / Workplace / Job seeking / Volunteer work / Public authorities / Family / Housing renting/buying / Banking / Healthcare / Elderly care / Shop/restaurant / Local community / Other |
| 76. Who discriminated against you? (Check all that apply.) | Work colleagues / Other ethnic group / Same ethnic group / Public employee / Healthcare personnel / Family/relatives / Classmates/students / Teachers / Friends / Unknown |
| 77. Have you been subjected to any form of physical violence? | Yes, as a child / Yes, as an adult / No, never |
| 78. If yes, by whom? | Stranger / Family / Spouse/partner / Other acquaintances |
| 79. Has anyone systematically tried to subdue, control, humiliate, insult, or degrade you? | Yes, as a child / Yes, as an adult / No, never |
| 80. If yes, by whom? | Stranger / Family / Spouse/partner / Other acquaintances |
| 81. Have you been subjected to any form of sexual abuse? | Yes, as a child / Yes, as an adult / No, never |
| 82. If yes, who? | Stranger / Family / Spouse/partner / Other acquaintances |
| 83. How do you rate your dental and oral health? | Very good / Quite good / Neither good nor bad / Quite bad / Very bad |
| 84. When was your last visit to the dentist/dental hygienist? | < 1 year ago / 1–2 years ago / 3–5 years ago / > 5 years ago |
| 85. If >2 years, why? (Check all that apply.) | No dental needs / Not called / No time / Long waiting time / Economic reasons / Fear / Transport issues / Health problems / Other |
| 86. In the last 2 years, have you received any dental diagnosis? | Yes / No / Don’t know: Cavities / Gum inflammation / Periodontal disease / Bruxism / Root canal treatment / Tooth fracture / Dry mouth / Other |
| 87. How were you treated at your last dental visit? | Very good / Quite good / Neither good nor bad / Quite bad / Very bad |
| 88. Overall, how satisfied are you with dental care in your municipality? | Very satisfied / Satisfied / Neither satisfied nor dissatisfied / Dissatisfied / Very dissatisfied |
| 89. In the last 12 months, have you been in contact with healthcare for your health? | Yes / No |
| 90. What type of healthcare contact did you have? (Check all that apply.) | District nurse / Doctor at primary health care center / Doctor at hospital / 1177 health care guide/ Counselor/Psychologist / Physiotherapist /Occupational therapist / Private provider / Digital provider / Other |
| 91. Did the healthcare contact concern the Covid‑19 pandemic? | Yes / No |
| 92. In the last 12 months, have you received care later than needed due to long waiting times? | Yes / No / I have not needed care |
| 93. In the last 12 months, have you received care later than needed due to distance/transport issues? | Yes / No / I have not needed care |
| 94. In the last 12 months, have you needed but couldn’t afford any of the following? | Medical care / Prescription medication / Psychological treatment |
| 95. Overall, how satisfied are you with healthcare in your municipality? | Very satisfied / Satisfied / Neither satisfied nor dissatisfied / Dissatisfied / Very dissatisfied |
| 96. If you’ve had healthcare contact in the last 12 months, how well do these apply? | Disagree completely (1) to Agree completely (5): • Staff listened • Staff spoke understandably • I was involved • I gained confidence • I am satisfied |
| 97. At your last healthcare visit, what language did you speak with the staff? | Swedish / Sami / Other (specify): ____ |
| 98. What language do you prefer to speak with healthcare staff? | Swedish / Sami / Other (specify): ____ |
| 99. If answered a language other than Swedish in Q98, were you offered an interpreter? | Yes / Do not wish to use an interpreter / No |
| 100. If an interpreter was used, who interpreted? | Authorized interpreter / Staff / Family/friend / Other |
| 101. Have you ever experienced not receiving interpreter assistance despite requesting it? | Yes / No |
| 102. Have you visited an alternative practitioner in the last 12 months? | Yes / No |
| 103. If yes, what type of treatment did you receive? (Check all that apply.) | Acupuncture / Healer / Homeopathy / Chiropractic / Massage / Naprapathy / Zone therapy / Herbs / Other |
| 104. Why do you use alternative medicine? (Check all that apply.) | Lack of effect from treatment / Avoid side effects / Complement to treatment / To try all possibilities / Did not get medical treatment / To prevent illness / Belief in alternative medicine / Past experiences / Recommended by healthcare / Recommended by others / Other |
| 105. How often do you eat vegetables/fruit/berries? | Most of year: Twice a day or more / Once a day / A few times a week / Once a week or less |
| 106. How often do you eat fish/seafood as a main course? | Three times a day or more / Twice a week / Once a week / A few times a month or less |
| 107. How often do you eat meat dishes/reindeer/elk snacks; pastries, chocolate/candy, chips or soda/juice? | Twice a day or more / Daily / A few times a week / Once a week or less |
| 108. How often do you eat breakfast? | Daily / Almost every day / A few times a week / Once a week or less |
| 109. Which breakfast option best matches yours? | Coffee/tea + sandwich / Coffee/tea + sweet bun/rusks / Filmjölk with cereal ± sandwich / Porridge ± sandwich / Gruel ± sandwich / I do not eat breakfast / None of the above |
| 110. Do you usually eat lunch? | Yes / No |
| 111. Do you usually eat dinner? | Yes / No |
| 112. Is there anything else you would like to convey that we have not asked about? | Free text |
