# Supplementary material for: Protocol for the HALDI study—conceptual framework for investigating health and living conditions in an arctic area of Sweden with a multiethnic population
Source: Ann Med. 2025 Jul 28;57(1):2537914. doi: 10.1080/07853890.2025.2537914 (PMC12308873; doi:10.1080/07853890.2025.2537914)
Supplement: Supplementary Table S2.docx [file IANN_A_2537914_SM0814.docx]

# Supplementary Table S2. Questionnaire HALDI study 2022

| **Question** | **Reply Options** |
| --- | --- |
| 1. Consent: I consent to participate in the health examination in accordance with the written information I have received in the research participant information. | Yes / No |
| 2. Consent: I consent to my survey responses being linked with national register data such as Statitistics Swedens’s business register, the pharmaceutical register, and the national patient register. | Yes / No |
| 3. Background: What year were you born? | Year (YYYY) |
| 4. Which best describes your current marital status? | Unmarried Married/registered partnership/cohabiting Divorced/previously registered partnership/separated Widow/widower/surviving partner Other marital status |
| 5. Living arrangement: Fill in the option that suits you. | Lives alone Lives with children/grandchildren Lives with partner Other, specify: _____ |
| 6. Accommodation: Type of housing: | House Assisted living facility or equivalent Group home Senior housing/sheltered housing Apartment (not service house) Nursing home Other, specify: _____ |
| 7. Are you satisfied with your current accommodation? | Yes / No |
| 8. School and Work: How many years have you attended school or engaged in full-time studies? | _____ years |
| 9. Have you changed your occupation during the last year? | Yes No (If no, go to question 11) |
| 10. What is your current occupation? | Employed On leave or parental leave Student, intern Unemployed In labor market measure Old-age pensioner Early retirement pensioner Permanently disabled, sickness pensioner Long-term sick leave (more than 3 months) Other - non-gainful employment  I am Employed I am Self-employed |
| 11. My employment rate is: | _____ % |
| 12. How often or always do the following describe your work? | Very often/always;Quite often;Sometimes;Quite rarely;Very rarely/never: • Work physically demanding • Work very sedentary • Need to lift heavy objects • Repetitive / monotonous movements • Exposed to strong shaking/vibrations • Work mentally demanding • Time for recovery • Experience stress |
| 13. Tobacco: Do you currently smoke cigarettes? | Yes, regularly (≥1 cigarette per day) Sometimes (< 1 cigarette per day) No |
| 14. Approximately how many cigarettes do you smoke on average per day? | _____No of cigarettes |
| 15. How many years have you smoked? | _____No of years |
| 16. Have you ever smoked cigarettes regularly before? | Yes, regularly (1 cigarette or more per day) No |
| 17. What year did you stop smoking cigarettes regularly? | YYYY |
| 18. How old were you when you started smoking cigarettes? | ___years |
| 19. Have you ever used snus? | Yes, less than 2 cans per week Yes, 2-4 cans per week Yes, more than 4 but less than 7 cans per week Yes, 7 cans or more per week Yes, used previously but not now No |
| 20. How many years ago did you stop using snus? | _____No of years |
| 21. How many years have you used snus? | _____No of years |
| 22. Gambling Habits: Have you ever, during the last 12 months, bought lottery tickets or gambled for money? | Yes No (Go to question 22) |
| 23. In the last 12 months, have you needed to gamble with larger sums to get the same feeling of excitement? | Never / Sometimes / Often / Almost always |
| 24. ...gambled for more than you could really afford to lose? | Never / Sometimes / Often / Almost always |
| 25. ...returned another day to try to win back the money you lost? | Never / Sometimes / Often / Almost always |
| 26. ...borrowed money or sold something to have money to gamble for? | Never / Sometimes / Often / Almost always |
| 27. Health - Quality of Life: How do you assess your general state of health? | Very good / Good / Neither good nor bad / Bad / Very bad |
| 28. How do you assess your mental health to have been in the last year? | Very good / Good / Neither good nor bad / Bad / Very bad |
| 29. Walking | I have no problems walking I have some problems walking I am bedridden |
| 30. Personal hygiene | I have no problems with my personal hygiene I have some problems with washing and dressing myself I cannot wash myself |
| 31. Daily activities | I have no problems performing daily activities I have some problems performing daily activities I cannot perform my daily activities |
| 32. Pain and discomfort | I have no pain or discomfort I have moderate pain and discomfort I have severe pain and discomfort |
| 33. Anxiety and depression | I am neither anxious nor depressed I am somewhat anxious and depressed I am very anxious and depressed |
| 34. On this scale, mark how good or bad your health condition is (0-100) | Fill in the number here: _____ |
| 35. Over the past 2 weeks, how often have you been bothered by the following problems? | Not at all/Several days/More than half of the days/Almost every day  Little interest or pleasure in doing things Feeling down, depressed, or hopeless Feeling nervous, anxious, or very stressed Unable to stop or control worrying |
| 36. If you checked any problems in question 35, how much difficulty have these problems caused you? | No difficulty at all / Some difficulty / Great difficulty / Extreme difficulty |
| 37. Feelings and thoughts during the past month | Response options: Never/Quite rarely/Sometimes/ Quite often/Very often:  -Unable to control important things  -Able to handle personal problems -Felt things were going your way - Felt difficulties were piling up to the point where they are unmanageable |
| 38. In the last year, have you been diagnosed by a doctor with any of these medical conditions? | Type 1 Diabetes / Type 2 Diabetes / Hypertension / Hyperlipidemia / Myocardial Infarction / Angina Pectoris / Heart Failure or Other Cardiac Disease / Stroke / Asthma / Allergies Requiring Treatment / Chronic Bronchitis, Emphysema, COPD / Rheumatic Disease / Fibro-myalgia / Chronic Pain / Osteoporosis / Osteoarthritis / Metabolic Disorders / Parkinson’s Disease / Multiple Sclerosis (MS) / Dementia or Memory Disorder / Gastrointestinal Disease / Cancer / Sleep Apnea / Gluten Intolerance / Lactose Intolerance / Milk Allergy / None of the Above |
| 39. Have you previously undergone bypass surgery or balloon angioplasty of the coronary arteries? | Yes / No |
| 40. Are you currently taking any prescription medication? | Yes, state how many: _____ No (Go to question 42) |
| 41. Which medication(s) do you take regularly? (Check all that apply.) | Oral antidiabetic / Insulin / Antihypertensive / Lipid-lowering / Cardiac or angina medication / Anticoagulants / Asthma or COPD medication / Corticosteroids or anti-inflammatory / Ulcer medication / Cancer treatment drugs / Metabolic disorder medications / Hormonal therapy / Analgesics / Antidepressants / Sedatives / Hypnotics / Herbal or natural remedies / Dietary supplements / Other |
| 42. Have you had any aches or pain in the last week? | Yes / No |
| 43. Do you know what the ache/pain is due to? | Yes / No |
| 44. Are you taking any medication for the ache/pain? | Yes / No |
| 45. If yes to question 44: does it help? | - Very good - Quite good - Quite bad - Not at all |
| 46. How has your ache/pain been in the last week? (Scale 0-10) | Mark one of the boxes below:  No ache/pain (0) - Worst imaginable pain (10) |
| 47. Do you get pain - tingling - chest pain when walking uphill or up stairs, or when walking fast on flat ground? | Yes / No |
| 48. Do you get pain - tingling - chest pain when walking at a normal pace on flat ground? | Yes / No |
| 49. If you get pain or discomfort in your chest when you move, what do you usually do? | - Stop - Slow down - Continue at the same pace |
| 50. If you stop or slow down, how long does it take for the pain to disappear? | - Immediately - After less than 10 minutes - After more than 10 minutes - The pain remains for a long time |
| 51. Have you ever had severe chest pain that lasted for half an hour or more? | Yes / No |
| 52. Do you usually get calf pain when walking uphill or on flat ground? | Yes / No |
| 53. Do you get short of breath from walking up two flights of stairs or equivalent at the same pace as people your age? | Yes / No |
| 54. Have you had wheezing or whistling in your chest at any time during the last 12 months? | Yes / No |
| 55. Have you been even slightly out of breath when you had this wheezing or whistling sound in your chest? | Yes / No |
| 56. Have you had this wheezing or whistling in your chest when you were not also suffering from a cold? | Yes / No |
| 57. Do you cough or clear phlegm (or have phlegm that is difficult to get up, despite coughing) most days for periods of at least 3 months per year? | Yes / No |
| 58. State for how many years you have had this problem: | ____ (Number of years) |
| 59. Do you wake up at night due to shortness of breath? | - Yes, every night - Yes, once a week or more - Yes, less than once a week (1-3 times/month) - No |
| 60. Has or did your father, mother, or siblings or children have diabetes? | Yes / No / Don’t know |
| 61. If yes, mark all applicable: | Yes, my mother / Yes, my father / Yes, one or more siblings / Yes, one or more children |
| 62. Has or did your father, mother, or siblings or children have chronic bronchitis, chronic obstructive pulmonary disease (COPD) or emphysema? | Yes / No / Don't know |
| 63. If yes, mark all applicable: | Yes, my mother / Yes, my father / Yes, one or more siblings / Yes, one or more children |
| 64. Has your father, mother, or siblings fallen ill/died from a heart attack (blood clot in the heart)? | Yes / No / Don't know |
| 65. If yes, mark all applicable: | Yes, my mother / Yes, my father / Yes, one or more siblings |
| 66. How old was your mother then? (State approximate age) | _____ |
| 67. How old was your father then? (State approximate age) | _____ |
| 68. Has your father, mother or siblings fallen ill/died from stroke (cerebral attack/brain infarction/blood clot in the brain/brain hemorrhage)? | Yes / No / Don't know |
| 69. If yes, mark all applicable: | Yes, my mother / Yes, my father / Yes, one or more siblings |
| 70. How old was your mother then? (State approximate age) | _____ |
| 71. How old was your father then? (State approximate age) | _____ |
| 72. Has or did your father, mother, siblings or children have any neurological disease, e.g., Parkinson's disease, Multiple Sclerosis (MS), Memory disorder such as Alzheimer's dementia or other type of dementia? (If yes, mark all applicable) | Yes, my mother / Yes, my father / Yes, one or more siblings / Yes, one or more children |
| 73. How old was your mother then? (State approximate age) | _____ |
| 74. How old was your father then? (State approximate age) | _____ |
| 75. How do you rate your dental health? | Very good / Quite good / Neither good nor bad / Quite bad / Very bad |
| 76. In the last three months, have you considered yourself to be in need of dental care but refrained from seeking care? | Yes / No (Go to question 78) |
| 77. What was the reason or reasons why you did not seek dental care? (Multiple options can be selected) | I have not had dental needs / I have not been called / I have not had time / There is a long waiting time / Economic reasons / I am afraid to go to the dentist/dental hygienist / Problems with transportation/long travel distance / Due to health problems / Other reason |
| 78. In the last 2 weeks, have you had any difficulties falling asleep? | None / Mild / Moderate / Severe / Very severe |
| 79. In the last 2 weeks, have you had any difficulties sleeping through the night? | None / Mild / Moderate / Severe / Very severe |
| 80. In the last 2 weeks, have you had any problems with waking up too early? | None / Mild / Moderate / Severe / Very severe |
| 81. How satisfied/dissatisfied are you with your current sleep pattern? | Very satisfied / Satisfied / Neither satisfied nor dissatisfied / Dissatisfied / Very dissatisfied |
| 82. To what extent do you think your sleep problems interfere with your life (your work, activities, concentration, memory, mood, etc.)? | Not at all disruptive / A little / Moderately / Much / Very much |
| 83. How noticeable to others do you think your problem with reduced sleep quality is? | Not at all noticeable / A little / Moderately / Much / Very much |
| 84. How worried or bothered are you about your sleep problem? | Not at all worried / A little / Moderately / Much / Very much |
| 85. Do you snore when you sleep? | Yes, always / Yes, almost always / Yes, sometimes / No, almost never / No, never / Don't know if I snore |
| 86. Have you been diagnosed with sleep apnea by a doctor? | Yes / No (Go to question 89) |
| 87. Do you have any treatment for sleep apnea? | Yes / No (Go to question 89) |
| 88. What type of treatment do you currently have? | CPAP / Bite splint / Surgery in the pharynx / Other |
| 89. How much time do you spend on everyday exercise in a typical week? (Everyday exercise = for example, walking, cycling or housework. Add up all time (at least 10 min at a time)) | 0 minutes/no time / < 30 minutes per week / 30-60 minutes per week / 60-90 minutes per week / 90-150 minutes per week / 150-300 minutes per week / > 300 minutes per week |
| 90. How much time do you spend on physical training that makes you short of breath in a typical week? (Physical training = for example, running, aerobic gymnastics or ball sports.) | 0 minutes/no time / < 30 minutes per week / 30-60 minutes per week / 60-90 minutes per week / 90-150 minutes per week / 150-300 minutes per week / > 300 minutes per week |
| 91. Mark in the table below how you usually travel to and from work/studies each season: | Car / Bus, train, boat / Walks / Cycling (for Spring, Summer, Autumn, Winter) |
| 92. How many km is it to your workplace? (one way) | _____ |
| 93. Mark the option that best describes your work: | Sedentary or standing / Light but partly mobile / Light and mobile / Sometimes physically demanding / Physically demanding most of the time |
| 94. How often have you exercised or trained in workout clothes in the last three months, with the aim of improving your fitness and/or feeling good? | Never / Occasionally / 1-2 times/week / 2-3 times/week / > 3 times/week |
| 95. How much have you moved and exerted yourself physically in your free time during the last 12 months? | Sedentary leisure time / Some physical activity in leisure time for at least 4 h per week / Regular moderate physical activity and training for at least 2-3 h per week / Regular hard training and competitive sport |
| 96. I am convinced that I have or have had a viral infection caused by the new Coronavirus. | Strongly disagree / Partially agree / Strongly agree |
| 97. I feel that the Covid-19 pandemic has affected my mental health. | For the better / Unchanged / For the worse |
| 98. The Covid-19 pandemic has affected my physical health. | For the better / Unchanged / For the worse |
| 99. The Covid-19 pandemic has affected my financial situation. | For the better / Unchanged / For the worse |
| 100. The Covid-19 pandemic has affected my social situation. | For the better / Unchanged / For the worse |
| 101. As a result of the Covid-19 pandemic, have you experienced any of the following? (For each, select one: Yes, No, Not relevant) | Significantly increased workload / Temporary lay-off / Unemployment / Sick leave |
| 102. The government has recommended social distancing and working from home to reduce the spread of the new Coronavirus. How easy has it been for you to follow this recommendation? | Very easy / Quite easy / Somewhat difficult / Very difficult / My work cannot be performed from home / Not relevant |
| 103. How do you feel that your physical work environment (e.g., ergonomics, light, air) has been affected by you working from home? | It has improved / It is unchanged / It has deteriorated / Not relevant |
| 104. How do you feel that your organizational psychosocial work environment (e.g., demands, control over work, social support) has been affected by you working from home? | It has improved / It is unchanged / It has deteriorated / Not relevant |
| 105. To what extent has the Covid-19 pandemic affected your physical activity? (Physical activity means both exercise/training and everyday exercise such as walking and cycling). | It has decreased / Unchanged / It has increased |
| 106. Have your commuting habits to and from work changed during the Covid-19 pandemic? | Yes / No |
| 107. In what way have your commuting habits changed? | I have reduced my total commuting time to and from work / My total commuting time to and from work is unchanged / I have increased my total commuting time to and from work |
| 108. Do you believe that the part of your daily exercise that previously consisted of transportation to and from work has been negatively affected by the Covid-19 pandemic? | Yes / No |
| 109. To what extent has the Covid-19 pandemic affected your possible use of? (For each, select one: It has decreased, Unchanged, It has increased, Do not use) | Cigarettes / Snus / Alcohol |
| 110. Due to the Covid-19 pandemic, have you avoided seeking care for something you would normally have sought care for? | Yes / No, I have sought care as usual / Have had no reason to seek care |
| 111. For what did you avoid seeking care? | _____ |
| **If you are younger than 65 years, go to question 125.** |  |
| 112. At what age did you retire? | ____Number of years_ |
| 113. Do you have a personal alarm? | Yes / No |
| 114. Do you have access to special transport service? | Yes / No |
| 115. Is your home designed so that you can do what you want and need to? | Yes / No |
| 116. If no, what is missing? | _____ |
| 117. Have you received housing adaptations? | Yes / No |
| 118. Do you have assistive devices? | Yes / No |
| 119. Do you have the assistive devices that you feel you need? | Yes / No |
| 120. Do you use the assistive devices you have at home? | Yes / No |
| 121. Do you have any form of mobility aid? | Yes / No |
| 122. If yes, which of your mobility aids do you use? (Check all that apply) | Wheelchair / Electric wheelchair / Cane/crutch / Walking poles / Walking frame / Rollator / Booster cushion / Kick scooter for outdoor use / Other, specify: _____ |
| 123. Have you changed your residence in the last year? | Yes / No |
| If yes, what type of residence have you moved to? | Elderly care home / Service apartment / Nursing home / Dementia care home |
| 124. Do you have any other support measures at home? | Yes / No |
| If yes, check below. | Home care service / Home healthcare / Day activity center / Help with medication dispensing / Food delivery / Help from relatives |
| 125. Mark the type of food you eat: | - I eat most things (mixed diet) - I eat fish, but not meat - I eat vegetarian food with dairy products and eggs (lacto-ovo vegetarian) - I eat vegetarian food with dairy products but not eggs (lacto-vegetarian) - I eat vegetarian food with eggs but not dairy products (ovo-vegetarian) - I eat vegan food |
| 126. Do you eat any of the following special diets (apart from what you indicated in the previous question)? | - I do not eat any special diet - I eat lactose-free diet - I eat completely dairy-free diet - I eat gluten-free diet - I eat other special diet |
| 127. Which special diet? | ________ |
