# Supplementary material for: Protocol for the HALDI study—conceptual framework for investigating health and living conditions in an arctic area of Sweden with a multiethnic population
Source: Ann Med. 2025 Jul 28;57(1):2537914. doi: 10.1080/07853890.2025.2537914 (PMC12308873; doi:10.1080/07853890.2025.2537914)
Supplement: Supplementary Table S3.docx [file IANN_A_2537914_SM0813.docx]

**Supplementary Table S3. Laboratory methods.**

| Test | Method | Sample Material | Reference Interval |
| --- | --- | --- | --- |
| Hemoglobin (Hb) | Colorimetric method using sodium lauryl sulfate | EDTA tube | Men: 130–170 g/L; Women: 117–153 g/L |
| Erythrocytes (RBC) | Automated flow cytometry/impedance | EDTA tube | Men: 4.2–5.8 ×10⁹/L  Women: 3.8–5.2 ×10⁹/L |
| Leukocytes (WBC) | Automated flow cytometry/impedance | EDTA tube | 3.5–8.5 ×10⁹/L |
| EVF (Hematocrit) | Automated measurement (calculated from Hb/MCV) | EDTA tube | Men: 0.39–0.50  Women: 0.36–0.46 |
| Mean Corpuscular Volume (MCV) | Automated flow cytometry/impedance | EDTA tube | 82-98 fL |
| Mean Corpuscular Hb Concentration (MCHC) | Automated flow cytometry/impedance | EDTA tube | 317–357 g/L |
| Thrombocytes (Platelets) | Automated flow cytometry/impedance | EDTA tube | 150–400 ×10⁹/L |
| Iron | Spectroscopic method | Serum, gel tube | 9–34 µmol/L |
| Transferrin | Immunoturbidimetry | Serum, gel tube | Adults: 1.94–3.26 g/L |
| Transferrin Saturation | Calculated: (Iron ÷ (Transferrin × 1.25)) × 100% |  | Adults: 0.15–0.60  women: 0.10–0.50 |
| Ferritin | Chemiluminescent immunoassay | Serum, gel tube | Women: 8–228 µg/L; men: 20–318 µg/L |
| HbA1c | Latex agglutination turbidimetry / HPLC / IFCC enzyme | EDTA tube | 31-46 mmol/mol |
| Total-/HDL-/LDL- Cholesterol | Direct enzymatic spectrophotometry | Serum, gel tube | Total Chol <5.0 mmol/L  LDL 2.0-5.3  HDL >1.0 |
| Triglycerides | Glycerol phosphate oxidase method | Serum, gel tube | <2.6 mmol/L |
| Apolipoprotein A1 & B | Immunoturbidimetry/nephelometry | Serum, gel tube | Apo A1: ~1.2–2.0 g/L  Apo B: ~0.6–1.3 g/L |
| CRP | Immunoturbidimetry / immunochemical | Serum/plasma | <5 mg/L |
| Vitamin B12 (Cobalamin) | Chemiluminescent immunoassay | Serum, gel tube | ~138–650 pmol/L |
| Folate | Chemiluminescent immunoassay | Serum, gel tube | <6.9 nmol/L |
| Cystatin C* | Immunoturbidimetry + automatic eGFR (CAPA formula) | Serum, gel tube | >65 y:> 60 mL/min/1,73m² body surface area  51-65 y: 60-110     " 18-50 y: 80-125    " |
| Ionized Calcium (Ca²⁺) | Ion-selective electrode (ISE) | Serum, on ice | 1.15–1.33 mmol/L |

* Estimated GFR based on cystatin C was calculated using the equations available at <https://www.egfr.se/>
